# Clinical Impact of Diagnostic Delay by Red Flags Assessment in Transthyretin Amyloid Cardiomyopathy

**DOI:** 10.1016/j.jacadv.2026.103200

**Published:** 2026-09-09

**Authors:** Manuela Iseppi, Michele Moretti, Teresa Maria Capovilla, Elisa Zaro, Giulia Barbati, Eleonora Bonvicini, Fabiola Morat, Massimiliano Marini, Roberto Bonmassari, Gianfranco Sinagra, Marco Merlo, Aldostefano Porcari

**Affiliations:** a Division of Cardiology, Ospedale Santa Chiara, Azienda Sanitaria Universitaria Integrata del Trentino, Trento, Italy; b Division of Cardiology, Ospedale Santa Maria Del Carmine, Azienda Sanitaria Universitaria Integrata del Trentino, Rovereto, Italy; c Center for Diagnosis and Treatment of Cardiomyopathies, Cardiovascular Department, Azienda Sanitaria Universitaria Giuliano-Isontina, University of Trieste, Trieste, Italy; d European Reference Network for Rare, Low Prevalence and Complex Diseases of the Heart-ERN GUARD-Heart, Trieste, Italy; e Biostatistics Unit, Department of Medical Sciences, University of Trieste, Trieste, Italy

**Keywords:** diagnostic delay, heart failure, prognosis, risk stratification, transthyretin amyloidosis

## Abstract

**Background:**

Diagnostic delay remains a major barrier to timely treatment in transthyretin amyloid cardiomyopathy (ATTR-CM). Although current approaches describe disease status at diagnosis, they do not capture the temporal evolution of disease manifestations preceding clinical recognition. The prognostic relevance of prediagnostic red flags and their timing remains poorly defined.

**Objectives:**

The objectives of the study were to characterize prediagnostic trajectories of ATTR-CM, quantify the median time from amyloidosis red-flag onset to diagnosis, and evaluate its association with survival.

**Methods:**

We retrospectively included consecutive patients with ATTR-CM from 2 referral centers in Italy. Red flags before diagnosis were assessed across cardiac, neurological, orthopedic, and renal domains. For each patient, the median time from red-flag onset to diagnosis was calculated. The primary outcome was all-cause mortality. The secondary outcome was cardiovascular (CV) mortality.

**Results:**

A total of 208 patients were included (median age 80 years; 83.7% male; 96.6% wild-type transthyretin amyloidosis). Red flags frequently emerged several years before diagnosis, with orthopedic and neurologic manifestations representing the earliest signals. In this contemporary cohort, diagnostic pathways were heterogeneous but converged mainly through cardiology referral (74.5%). A longer median time from red-flag onset to diagnosis was independently associated with a higher risk of all-cause mortality (adjusted HR: 1.07; 95% CI: 1.01-1.14; *P* = 0.049) and CV mortality (adjusted HR: 1.10; 95% CI: 1.02-1.18; *P* = 0.025).

**Conclusions:**

The median time from red-flag onset to ATTR-CM diagnosis is an independent predictor of all-cause and CV mortality. Enhancing early identification of noncardiac red flags across specialties may shorten the diagnostic pathway, facilitate earlier initiation of disease-modifying therapy, and ultimately improve clinical outcomes.

Transthyretin amyloid cardiomyopathy (ATTR-CM) is a clinically heterogeneous disease, ranging from slowly progressive late-onset forms to more rapidly evolving phenotypes.^1^^,^^2^ Wild-type ATTR predominantly affects older adults and usually presents with cardiomyopathy, whereas hereditary ATTR, caused by pathogenic *TTR* variants, may manifest with variable combinations of cardiomyopathy and neuropathy.^3^^,^^4^ Survival is strongly influenced by the severity of cardiac involvement at diagnosis.

Although renewed disease awareness coupled with noninvasive diagnostic algorithm and the development of disease-modifying treatments have transformed the contemporary landscape of ATTR-CM, diagnostic delay remains a major challenge with significant impact on clinical management and disease evolution. ATTR-CM is often misdiagnosed with a median of 17 hospital attendances per patient to reach the correct diagnosis.^5^ Nearly half of patients experience diagnostic delays of more than 4 years,^5^ and longer time to diagnosis has been associated with worse survival.^6^ Registry data also suggest that nearly half of patients are diagnosed only after the onset of advanced heart failure (HF), when the benefit of disease-modifying treatment may be attenuated.^7^

A key contributor to diagnostic delay is the nonspecific nature of early manifestations. Timely diagnosis depends on the recognition of typical red flags^8^ including a broad spectrum of cardiac, neurological, orthopedic, and extracardiac features that may precede the diagnosis of ATTR-CM by several years.^9^ Among these, carpal tunnel syndrome (CTS) has emerged as one of the earliest clinical markers of future ATTR-CM and may provide an actional finding for designing screening programs in the general population. However, limited data are available on the type, onset, and clinical significance of other red flags preceding the diagnosis of ATTR-CM.

In this study, we aimed to: 1) characterize the clinical domains and timing of onset of typical red-flag in patients ultimately diagnosed with ATTR-CM; and 2) evaluate the potential association between the time interval from red-flag onset to ATTR-CM diagnosis and survival.

## Methods

### Study population

Consecutive patients with confirmed wild-type or variant ATTR-CM diagnosed between January 2014 and December 2024 at 2 referral amyloidosis centers in Italy (Cattinara University Hospital, Trieste, and Santa Chiara Hospital, Trento) were retrospectively identified through institutional amyloidosis registries. Clinical, laboratory, and instrumental data were extracted from electronic medical records. Diagnosis of ATTR-CM was established on the basis of HF symptoms together with a characteristic echocardiogram or cardiac magnetic resonance study and either endomyocardial biopsy proof of TTR amyloid or Perugini grade 2 or 3 myocardial uptake on cardiac scintigraphy in the absence of either an abnormal serum free light chain ratio or a monoclonal immunoglobulin in the serum or urine by immunofixation.^10^ The TTR gene was sequenced in all patients as previously described.^11^ All echocardiographic parameters were measured according to standard international definitions.^12^ The National Amyloidosis Center (NAC) ATTR staging system (from stage 1-3) was defined on the basis of N-terminal pro–B-type natriuretic peptide (BNP) (NT-proBNP) (3000 ng/L) and estimated glomerular filtration rate (eGFR) (45 mL/min/1.73 m^2^).^13^ As our patients had a combination of BNP and NT-proBNP at baseline, the NAC ATTR stage was measured by applying a previously used conversion whereby a BNP cutoff >600 pg/mL corresponds to NT-proBNP >3000 pg/mL.^14^^,^^15^ The study was approved by the local ethics committees of the participating centers and conducted in accordance with the Declaration of Helsinki.

### Identification and classification of red flags

Prediagnostic clinical, laboratory, electrocardiographic, and imaging data were systematically reviewed to identify established red flags of ATTR-CM based on 2021 European Society of Cardiology Position Statement on Cardiac Amyloidosis,^9^ which were classified into 4 predefined domains using standardized criteria across participating centers. The domains included.

• Cardiac: interventricular septal thickness ≥12 mm, HF onset at ≥65 years, aortic stenosis at ≥65 years, persistent hypotension or normotension in previously hypertensive patients, chronically elevated troponin, pseudo-Q waves on surface electrocardiogram, reduced voltage-to-mass ratio, conduction system disease, apical sparing pattern on speckle-tracking echocardiography, late gadolinium enhancement, or increased extracellular volume on cardiac magnetic resonance.
• Neurological: autonomic dysfunction; sensory involvement; peripheral polyneuropathy.
• Orthopedic: bilateral CTS; biceps tendon rupture.
• Renal: proteinuria defined as abnormal urinary protein excretion (>150 min/24 h) and/or albuminuria >30 min/24 h (or mg/g creatinine).

Incidental cardiac uptake on bone scintigraphy performed for nonamyloid reasons was also collected as an exploratory nonclinical domain.

For each red flag, the interval between its first documentation and the date of ATTR-CM diagnosis was recorded. For each patient, the domain of the earliest red flag and the domain prompting referral to a specialized amyloidosis center were assessed. The median time from red-flag onset to ATTR-CM diagnosis was then calculated.

### Outcomes

The primary outcome was all-cause mortality. The secondary outcome was cardiovascular (CV) mortality. The mortality endpoint was defined as time to death from baseline for all deceased patients and time to censor date (December 31, 2025) from baseline among the remainder. CV mortality was defined as sudden cardiac death, aborted cardiac death, fatal stroke, fatal cardiac transplant, or end-stage HF with pump failure. Data were obtained from scheduled follow-up evaluations and electronic health records.

### Statistical analysis

Continuous variables are expressed as the median with 25th-75th percentiles (Q1-Q3) and compared using the Mann-Whitney *U* test. Categorical variables were summarized as counts and percentages and compared using the chi-square test or Fisher exact test, as appropriate. The associations between median time from red flag onset to ATTR-CM diagnosis and mortality were assessed using multivariable Cox proportional hazards models. Multivariable models were adjusted for age at diagnosis, sex, NAC stage, and tafamidis exposure. Model results are presented as the HR with 95% CI. Tafamidis exposure was modeled as a time-dependent covariate using a start-stop data structure. In this analysis, each individual not treated with tafamidis at the time of ATTR-CM diagnosis is identified as “not on treatment” from time 0 (ie, ATTR-CM diagnosis) to the day before initiation of tafamidis therapy and then is identified as “on treatment” until the end of observation (ie, death or censor date). This approach allows patients to contribute person-time both before and after treatment initiation. Effect modification by age at diagnosis (<80 vs ≥80 years) was assessed by including an interaction term. Time-dependent area under the receiver operating characteristic (ROC) curves (AUC) derived from the multivariable Cox models were estimated to assess model discrimination at 24 months from baseline; time-dependent AUCs were estimated using the inverse probability of censoring weighting approach (Uno method) implemented in the timeROC package. For graphical time-to-event analyses, patients were grouped into quartiles according to their median time from red-flag onset to diagnosis; for curve display, adjacent quartiles were combined into 2 groups (Q1-Q2 vs Q3-Q4). Kaplan-Meier and cumulative incidence analyses were performed using the date of ATTR-CM diagnosis as time zero. All-cause mortality was evaluated using Kaplan-Meier curves and compared with the log-rank test. CV mortality was evaluated using cumulative incidence functions and compared with Gray test. A competing-risk analysis considering non-CV mortality as a competing event was performed to explore the relationship between prediagnostic red flags and CV mortality.^16^ As a sensitivity analysis, time-to-event curves were additionally estimated after aligning follow-up according to treatment status, with time zero defined as tafamidis initiation in treated patients and ATTR-CM diagnosis in untreated patients. Kernel density estimation was applied to red-flag-specific time intervals to visualize the distribution and timing of manifestations across domains. To assess whether longer prediagnostic intervals primarily reflected a greater number of red flags, the association between the number of red flags and the median time from red-flag onset to diagnosis was examined using Spearman correlation coefficient (r) and graphical inspection. Transitions from the earliest red-flag domain to the domain prompting referral were visualized using a Sankey diagram weighted by the number of patients in each trajectory. All analyses were performed using R statistical software (version 2026.05.0 + 218; R Core Team, 2026). Time-to-event and competing-risk analyses were conducted using the tidycmprsk package, and time-dependent ROC curves were estimated using the timeROC package. All tests were 2-sided, and *P* < 0.05 was considered statistically significant.

## Results

### Study population

The study population was comprised of 208 patients with ATTR-CM. Most patients (n = 169, 81.3%) were diagnosed after 2020. The median age was 80 (Q1–Q3: 76-83) years, 83.7% were males and 96.6% had wild-type ATTR-CM. About 38.5% had atrial fibrillation, 79.8% had systemic hypertension, and 19.7% had ischemic heart disease. The median NT-proBNP was 1953 pg/mL (Q1–Q3: 1001-3761 ng/L) and median eGFR was 60 (Q1–Q3: 46-75) mL/min/1.73 m^2^). Most patients were in NAC disease stage 1 (59.5%) or 2 (29.5%), and 11.0% were in NAC disease stage 3 (Table 1). Patients presented with an high number of red flags at ATTR-CM diagnosis, with a median of 5 per patient (Q1–Q3: 4-6): 2 patients (1.0%) had 1 red flag, 9 (4.3%) had 2, 33 (15.9%) had 3, 36 (17.3%) had 4, 41 (19.7%) had 5, 45 (21.6%) had 6, 28 (13.5%) had 7, 10 (4.8%) had 8, 3 (1.4%) had 9, and 1 (0.5%) had 10 red flags.

**Table 1:** Baseline Clinical Characteristics of the Total Study Cohort

	Total Population (N = 208)	RF Median Time ≤9 Months (n = 110)	RF Median Time >9 Months (n = 98)	*P* Value
Age at diagnosis, y, median [Q1-Q3]	80 [76-83]	80 [76-83]	80 [76-83]	0.95
Male, n (%)	174 (83.7)	89 (80.9)	85 (86.7)	0.26
ATTR type				>0.99
ATTRwt, n (%)	201 (96.6)	106 (96.4)	95 (96.9)	
ATTRv, n (%)	7 (3.4)	4 (3.6)	3 (3.1)	
Ischemic heart disease, n (%)	41 (19.7)	19 (17.4)	22 (22.4)	0.37
Systemic hypertension, n (%)	166 (79.8)	84 (77.1)	82 (83.7)	0.23
Atrial fibrillation at diagnosis, n (%)	80 (38.5)	36 (32.7)	44 (44.9)	0.080
N of red flags per patient, median [Q1-Q3]	5 [4-6]	5 [4-6]	5 [4-6]	0.56
Median RF time before diagnosis, months, median [Q1-Q3]	9 [4-34]	–	–	
NT-proBNP at diagnosis, pg/mL, median [Q1-Q3]	1953 [1001-3761]	1855 [834-3761]	2114 [1305-3694]	0.13
eGFR, mL/min/m^2^, median [Q1-Q3]	60 [46-75]	60 [49-75]	60 [45-75]	0.82
NAC stage				0.49
NAC stage 1 at diagnosis, n (%)	113 (59.5)^a^	60 (61.9)^a^	53 (57.0)^a^	
NAC stage 2 at diagnosis, n (%)	56 (29.5)^a^	25 (25.8)^a^	31 (33.3)^a^	
NAC stage 3 at diagnosis, n (%)	21 (11.0)^a^	12 (12.3)^a^	9 (9.7)^a^	
Echocardiographic parameters at diagnosis				
IVS thickness, mm, median [Q1-Q3]	18 [15-20]	18 [16-20]	17 [15-19]	0.47
LVEF, %, median [Q1-Q3]	56.0 [48-63]	56.0 [50-64]	55.0 [46-63]	0.27
GLS, –%, median [Q1-Q3]	−12.9 [–10,–15]	−13.8 [–11,–15]	−10.9 [–8,–14]	**0.026**
TAPSE, mm, median [Q1-Q3]	19 [15-21]	19 [16-21]	18 [14-22]	0.24
sPAP, mm Hg, median [Q1-Q3]	39 [32-48]	38 [32-45]	43 [32-49]	0.25
Loop diuretic dose at diagnosis, mg, median [Q1-Q3]	25 [0-62.5]	25 [0-50]	50 [12.5-100]	**0.029**
Patients treated with tafamidis, n (%)	111 (53.4)	58 (53.7)	53 (54.1)	0.96
Time from diagnosis to tafamidis initiation, months, median [Q1-Q3]	4 [1-11]	4 [2-10]	4 [1-11]	0.90

**Bold** numbers indicate statistically significant values.

ATTRv = variant transthyretin amyloid cardiomyopathy; ATTRwt = wild-type transthyretin amyloid cardiomyopathy; eGFR = estimated glomerular filtration rate; GLS = global longitudinal strain; IVS = interventricular septal; LVEF = left ventricular ejection fraction; NAC = National Amyloidosis Center; NT-proBNP = N-terminal pro–B-type natriuretic peptide; RF = red flags; sPAP = systolic pulmonary artery pressure; TAPSE = Tricuspid Annular Plane Systolic Excursion.

a Percentages are calculated among patients with available data for NAC staging. Continuous variables are presented as median (Q1-Q3) and were compared using the Mann-Whitney *U* test. Categorical variables are presented as number (%) and were compared using the chi-square or Fisher exact test, as appropriate.

The median number of cardiac and noncardiac red flags was 4 (Q1-Q3: 3-5) and 1 (Q1-Q3: 0-2), respectively.

The overall number of red flags was not associated with markers of disease severity. Patients with ≥5 and <5 red flags (any type) had similar median NT-proBNP levels (1954 [Q1-Q3: 1109-4107] vs 1919 [Q1-Q3: 906-3645] pg/mL; *P* = 0.48), median eGFR (60 [Q1-Q3: 46-75] vs 60 [Q1-Q3: 46-75] mL/min/1.73 m^2^; *P* = 0.94), prevalence of NYHA functional class >II (12.6% vs 17.5%; *P* = 0.33) and NAC stage >1 (43.3% vs 35.7%; *P* = 0.30). Similar findings were observed when stratifying by number of cardiac red flags (≥4 and <4).

Compared with those with ≤1, patients with >1 noncardiac red flags exhibited lower median NT-proBNP levels (1503 [Q1-Q3: 767-2336] vs 2375 [Q1-Q3: 1214-4156] pg/mL; *P* = 0.006), with no differences in eGFR (60 [Q1-Q3: 48-78] vs 60 [Q1-Q3: 46-72] mL/min/1.73 m^2^; *P* = 0.39, NYHA functional class >II (15.9% vs 13.8%; *P* = 0.68), or NAC stage >1 (32.8% vs 44.4%; *P* = 0.12).

Tafamidis was initiated in 53.4% of patients at a median time of 4 months (Q1-Q3: 1-11) after ATTR-CM diagnosis (Table 1). Supplemental Table 1 shows baseline characteristics according to tafamidis exposure during follow-up. Patients receiving tafamidis were generally younger and presented with less advanced disease at diagnosis. Neither the number of red flags per patient nor the median time from red-flag onset to ATTR-CM diagnosis differed between treated and untreated patients.

### Temporal distribution of red flags before ATTR-CM diagnosis

Red flags showed a broad temporal distribution before diagnosis (Table 2). The most frequent red flags were HF onset at ≥65 years (73.6%) and bilateral CTS (49.5%). Neurological and orthopedic red flags, particularly peripheral polyneuropathy and bilateral CTS, were among the earliest manifestations and preceded diagnosis by a median of 80 months (Q1-Q3: 37-147) and 78 months (Q1-Q3: 40-123), respectively. Cardiac red flags were more closely distributed around the time of ATTR-CM diagnosis. Kernel density analysis showed distinct temporal distributions across red-flag domains, with earlier and broader distributions for orthopedic and neurological manifestations and a tighter clustering of cardiac red flags near diagnosis (Figure 1).

**Table 2:** Prevalence of Red Flags^9^ and Median Time From Onset to Diagnosis

Red Flags	Prevalence (n = 208)	Median [Q1, Q3] Time Before Diagnosis, Months
Interventricular septum ≥12 mm	208 (100.0%)	−4 [–43, 0]
Heart failure onset at age ≥65 years	153 (73.6%)	−10 [–85, −4]
Diagnosis of aortic stenosis at age ≥65 years	33 (15.9%)	−12 [–46, −3]
Subendocardial/transmural LGE or increased ECV	34 (16.3%)	−3 [–12, 0]
Apical sparing on global longitudinal strain	81 (38.9%)	−4 [–11, 0]
Reduced voltage-to-mass ratio	87 (41.8%)	−8 [–23, −2]
Pseudo-Q waves on ECG	47 (22.6%)	−7 [–29, −2]
AV conduction disease	66 (31.7%)	−8 [–28, −1]
Chronically elevated troponin	94 (45.2%)	−8 [–15, −4]
Hypotension or normotension in previously hypertensive	42 (20.2%)	−4 [–8, −1]
Autonomic dysfunction or sensory involvement	21 (10.1%)	−25 [–58, −9]
Peripheral polyneuropathy	62 (29.8%)	−80 [–147, −37]
Proteinuria	13 (6.3%)	−6 [–11, −3]
Bilateral carpal tunnel syndrome	103 (49.5%)	−78 [–123, −40]
Biceps tendon rupture	5 (2.4%)	−28 [–93, −26]

AV = atrioventricular; ECG = electrocardiogram; ECV = extracellular volume; LGE = late gadolinium enhancement.

**Figure 1:**
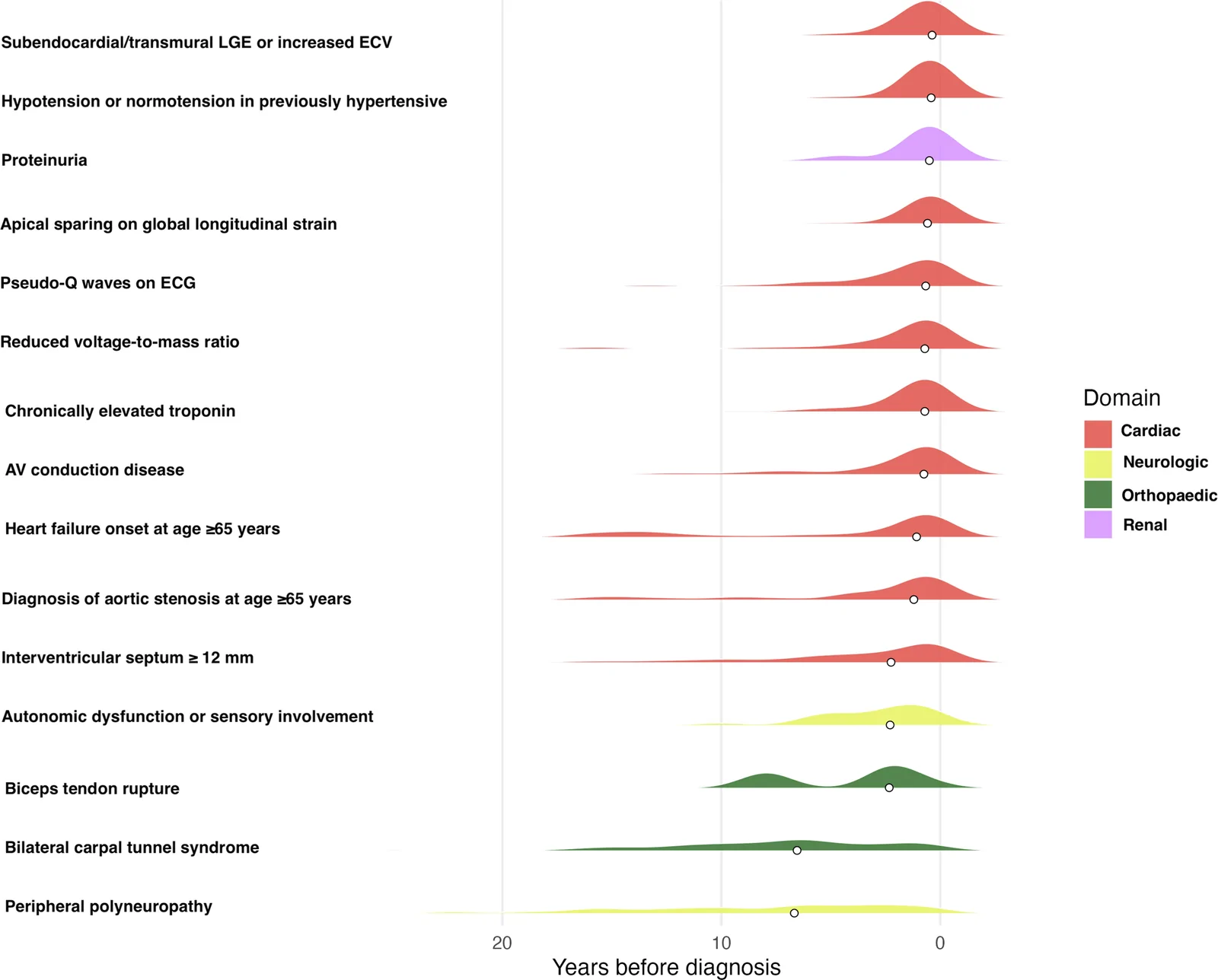
Temporal Distribution of Red Flags Before Diagnosis Kernel density plots showing the timing of individual red flags relative to ATTR-CM diagnosis across clinical domains. Orthopedic and neurological manifestations display broader and earlier distributions, whereas cardiac red flags cluster closer to diagnosis. White dots indicate median value. AV = atrioventricular; ECG = electrocardiogram; ECV = extracellular volume; LGE = late gadolinium enhancement.

Sankey flow analysis showed substantial shifts in diagnostic trajectories across clinical domains. Although cardiac red flags represented the earliest manifestation in nearly half of the cohort, their predominance increased further at referral, accounting for almost 3-quarters (74.5%) of pathways leading to referral to specialized amyloidosis centers. Neurological, orthopedic, and renal manifestations were frequently present early but triggered referral less often, indicating a progressive shift of referral pathways toward cardiology (Figure 2).

**Figure 2:**
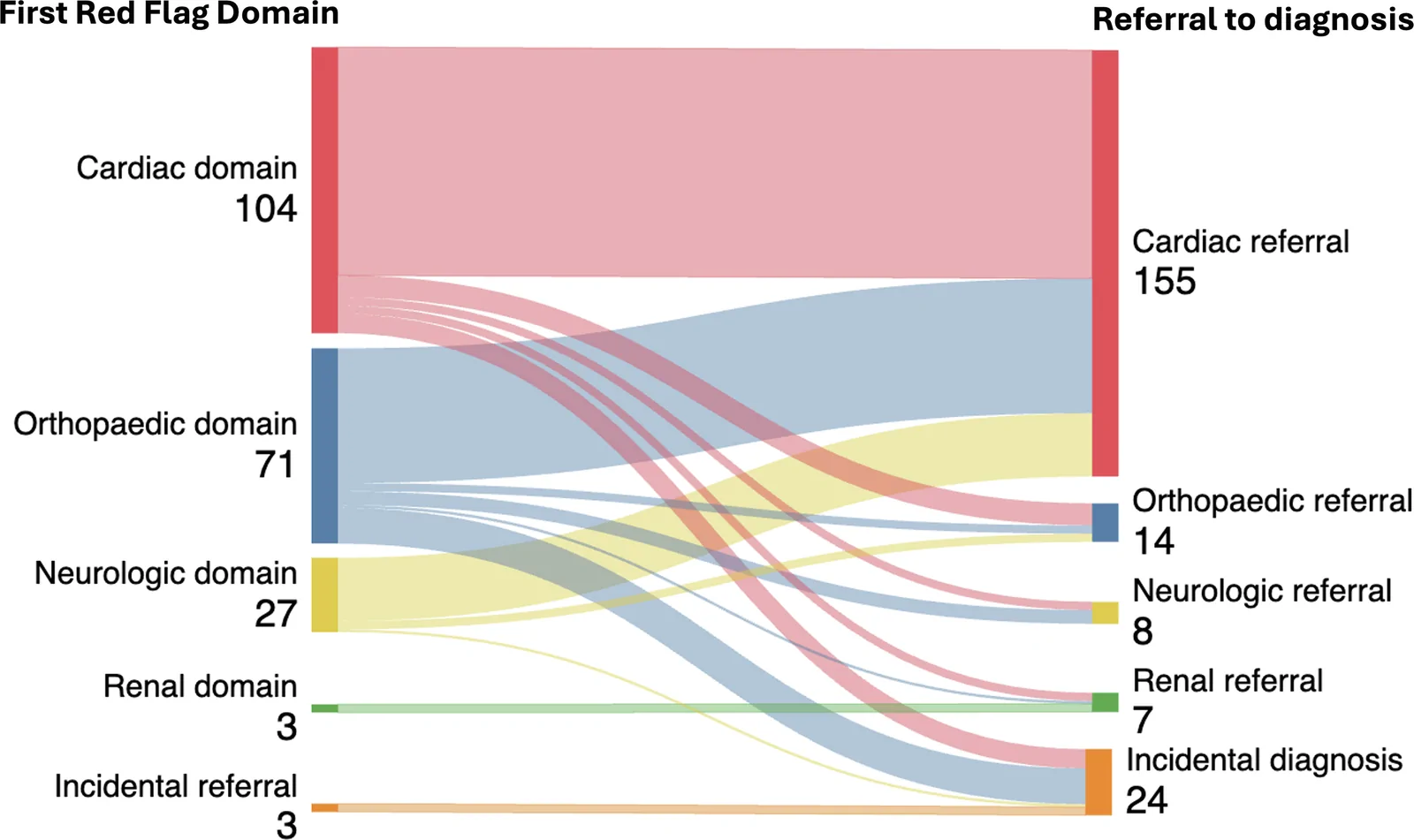
Diagnostic Pathways From Initial Presentation to Referral Sankey diagram illustrating patient trajectories from the earliest red-flag domain to the domain prompting referral to specialized amyloidosis centers. Flows are weighted by the number of patients, highlighting the progressive shift toward cardiology referral.

### Association between median time from red flag onset to diagnosis and survival

During a median follow-up of 27 months (Q1-Q3: 15-41), 62 patients experienced all-cause death and 48 had CV death.

Quartile cutoffs for the median time from red-flag onset to diagnosis were 4, 9, and 34 months. When patients were stratified into groups of early (Q1-Q2) vs late (Q3-Q4) diagnosis, those with longer diagnostic delay exhibited significantly higher rates of all-cause mortality (log-rank *P* = 0.047) and CV mortality (Gray test *P* = 0.014) (Figure 3). Findings were consistent in a sensitivity analysis using treatment-aligned follow-up, as shown in Supplemental Figure 1. In a multivariable Cox regression analysis with covariates age, sex, NAC disease stage, and tafamidis treatment, a longer time from red-flag onset to ATTR-CM diagnosis was independently associated with higher risk of all-cause mortality (HR: 1.07; 95% CI: 1.01-1.14; *P* = 0.049) and CV mortality (HR: 1.10; 95% CI: 1.02-1.18; *P* = 0.025) (Tables 3 and 4). Tafamidis treatment was independently associated with lower all-cause mortality (HR: 0.25; 95% CI: 0.11-0.56; *P* < 0.001). Model discrimination was good, with time-dependent AUCs of 0.85 (95% CI: 0.76-0.93) and 0.84 (95% CI: 0.74-0.94) for all-cause mortality and CV mortality at 24 months, respectively (Figure 4).

**Figure 3:**
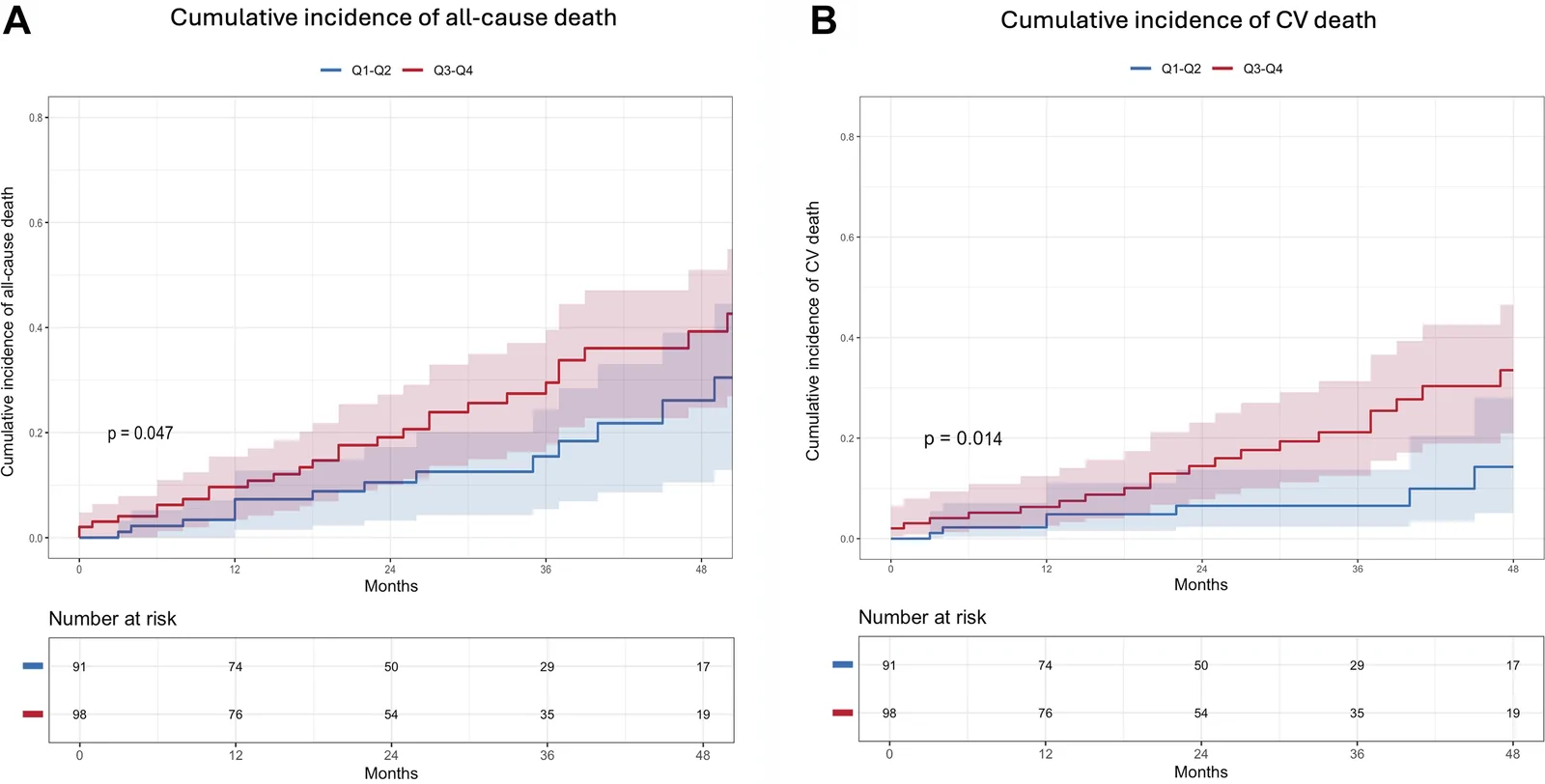
All-Cause and Cardiovascular Mortality by Diagnostic Delay Groups (A) Kaplan-Meier curves for all-cause mortality stratified by median time from red-flag onset to diagnosis (early vs late groups based on quartiles). Shaded areas represent 95% CIs; groups were compared using the log-rank test. (B) Cumulative incidence curves for cardiovascular mortality stratified by median time from red-flag onset to diagnosis, accounting for competing risks. Shaded areas represent 95% CIs; groups were compared using Gray test. Longer diagnostic intervals were associated with worse survival and higher cardiovascular mortality. CV = cardiovascular.

**Table 3:** Multivariable Cox Model for All-Cause Death

	HR	95% CI	*P* Value
Age at diagnosis	**1.08**	1.01-1.16	**0.019**
Female	0.47	0.17-1.33	0.12
Median RF time before diagnosis (year)	**1.07**	1.01-1.14	**0.049**
NAC stage at diagnosis	**1.71**	1.08-2.70	**0.022**
Tafamidis therapy	**0.25**	0.11-0.56	**<0.001**

**Bold** numbers indicate statistically significant values.

Abbreviations as in Table 1.

**Table 4:** Multivariable Cox Model for CV Death

	HR	95% CI	*P* Value
Age at diagnosis	1.08	0.99-1.17	0.067
Female	0.72	0.25-2.08	0.50
Median RF time before diagnosis (y)	**1.10**	1.02-1.18	**0.025**
NAC stage at diagnosis	**1.89**	1.09-3.26	**0.023**
Tafamidis therapy	**0.24**	0.09-0.62	**0.001**

**Bold** numbers indicate statistically significant values.

CV = cardiovascular; other abbreviations as in Table 1.

**Figure 4:**
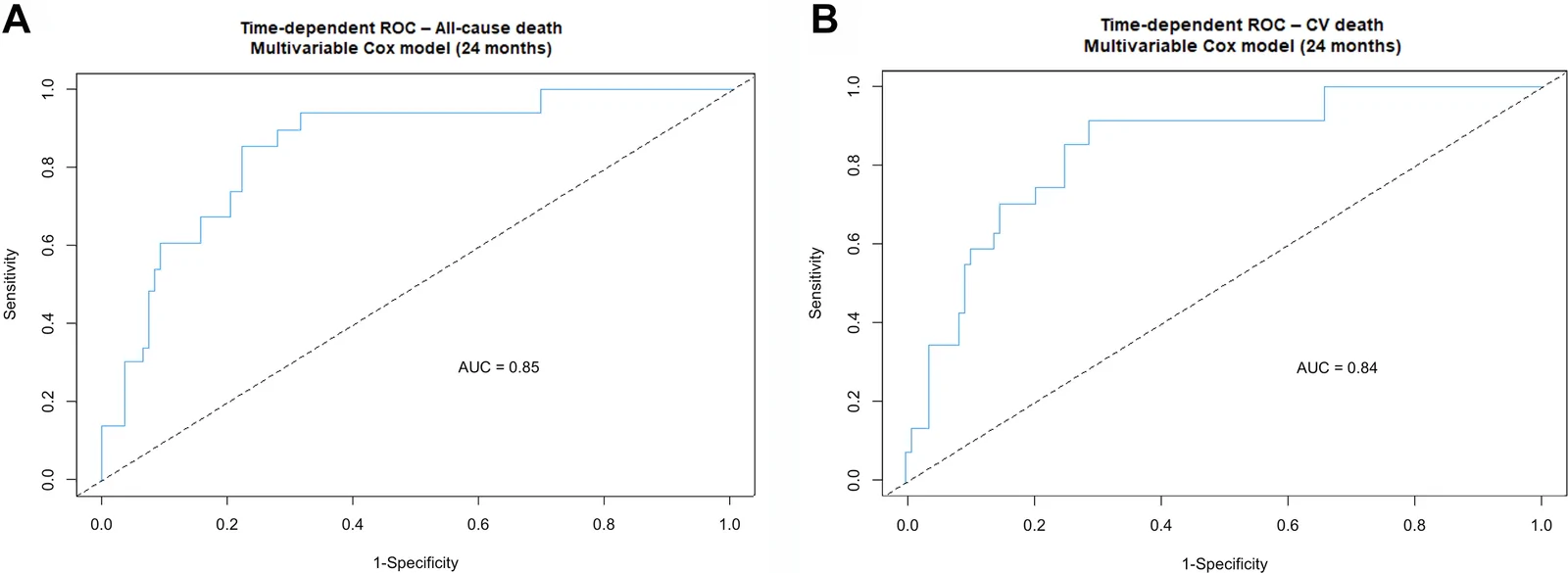
Time-Dependent ROC for All-Cause and Cardiovascular Mortality (A) Time-dependent ROC curves evaluating the predictive performance of the multivariable Cox model for all-cause mortality. (B) Time-dependent ROC curves assessing the predictive accuracy of the multivariable Cox model for cardiovascular mortality. The model includes median red-flag time, age, sex, NAC stage, and tafamidis treatment. AUC = area under the receiver operating characteristic curve; ROC = receiver operating characteristic; other abbreviation as in Figure 3.

The association between diagnostic delay (ie, median time from red flag onset to ATTR-CM diagnosis) and of all-cause mortality and CV mortality was consistent across age groups (<80 vs ≥ 80 years), with no significant interaction (*P* interaction = 0.58 for all-cause mortality; *P* interaction = 0.88 for CV mortality). The number of red flags at diagnosis was not associated with the median time from red-flag onset to ATTR-CM diagnosis (Spearman r = −0.09) (Figure 5).

**Figure 5:**
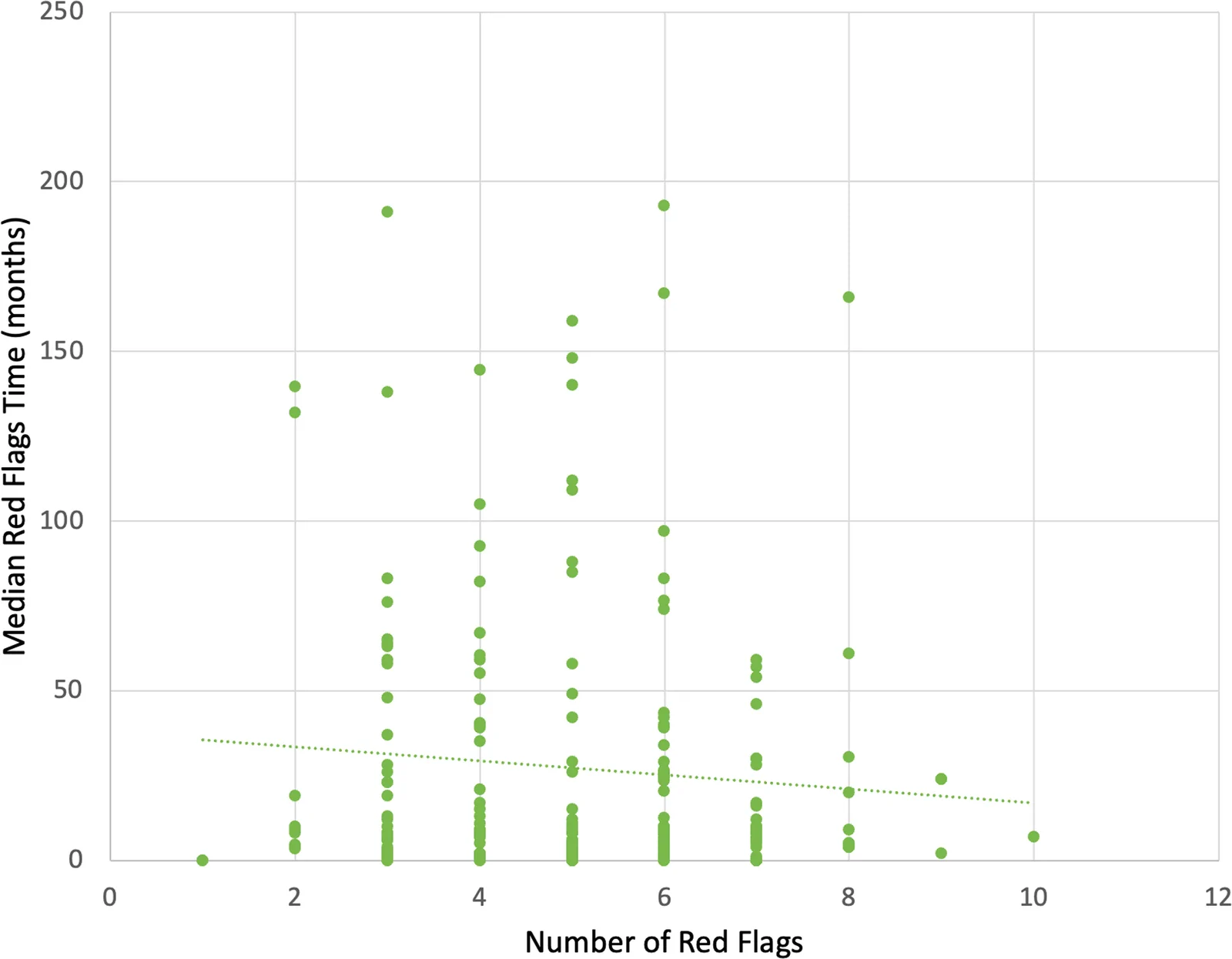
Red Flag Burden vs Diagnostic Delay Scatter plot showing the relationship between the number of red flags per patient and the median time from red-flag onset to ATTR-CM diagnosis. No significant correlation was observed (Spearman's r = −0.09).

## Discussion

This is the first study analyzing the type and timing of onset of a wide array of cardiac and noncardiac red flags before ATTR-CM diagnosis in a well-characterized contemporary cohort of patients with wild-type and hereditary ATTR-CM. This study provides information on the diagnostic course of ATTR-CM that is not captured by conventional clinical descriptions, and is the first to demonstrate the prognostic impact of an easily-accessible metrics, namely the median time from red-flag onset to ATTR-CM diagnosis, on survival in the full spectrum of disease. We found that: 1) patients with ATTR-CM present with a high burden of prediagnostic red flags, with orthopedic and neurological manifestations frequently preceding cardiac involvement by several years; 2) the overall number of red flags is not associated with markers of disease severity at diagnosis; 3) diagnostic pathway is characterized by a progressive shift from noncardiac to cardiac domains, with most patients ultimately reaching diagnosis through cardiology despite early extracardiac manifestations; and 4) a longer median time from red-flag onset to ATTR-CM diagnosis is independently associated with higher risk of all-cause mortality and CV mortality. Altogether, these findings indicate that overall diagnostic delay in recognizing red flags and achieving a definite diagnosis of ATTR-CM rather than the presence or number of individual red flags has important clinical and prognostic implication in ATTR-CM (Central Illustration).

**Central Illustration:**
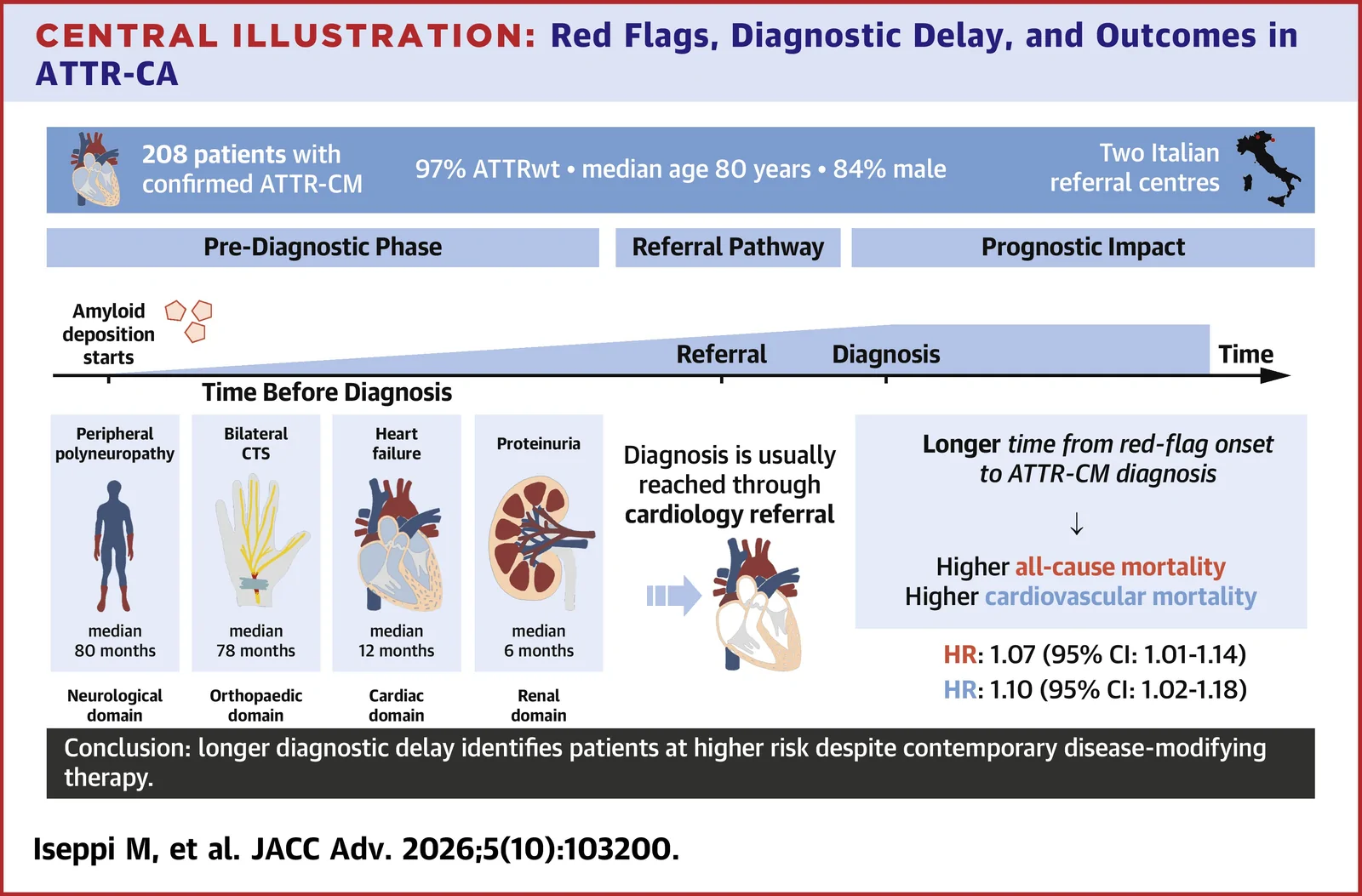
Red Flags, Diagnostic Delay, and Outcomes in ATTR-CA This illustration summarizes the temporal trajectory of transthyretin amyloid cardiomyopathy (ATTR-CM), from early extracardiac red flags to cardiac involvement and diagnosis. Orthopedic and neurological manifestations often precede diagnosis by several years, whereas cardiac red flags cluster closer to diagnosis and frequently drive referral to specialized centers. A longer interval between red-flag onset and diagnosis is associated with worse clinical outcomes, highlighting the importance of early recognition across specialties to reduce diagnostic delay and improve prognosis. ATTRwt = wild-type transthyretin amyloid cardiomyopathy; CTS = carpal tunnel syndrome.

ATTR amyloidosis may present with heterogeneous clinical phenotypes and rates of disease progression, with extracardiac manifestations preceding overt cardiac involvement by several years.^2^^,^^5^^,^^6^ Signs and symptoms of disease are nonspecific, contributing to significant diagnostic delay in clinical practice. Previous studies have mainly focused on symptom-to-diagnosis delay, reporting average intervals of 4 to 6 years,^5^^,^^17^, ^18^, ^19^ without distinguishing between early disease signals and delayed clinical recognition. They have not systematically assessed the type and time of onset of cardiac and noncardiac red flags, nor their association with outcomes. By systematically mapping prediagnostic cardiac and noncardiac red flags, our analysis provides more granular information.

In our cohort, red flags frequently emerged several years before ATTR-CM diagnosis, with orthopedic and neurological manifestations often preceding cardiac involvement. These findings are consistent with the multisystem nature of transthyretin deposition and align with previous studies reporting that conditions such as CTS may precede overt cardiac involvement by several years.^20^ In selected populations undergoing carpal tunnel surgery, ATTR-CM has been identified in a substantial proportion of older individuals with cardiac abnormalities such as left ventricular hypertrophy or HF signs and symptoms.^21^^,^^22^ In contrast, the onset of cardiac manifestations was more closely distributed around the time of ATTR-CM diagnosis. The relatively short interval between the onset of cardiac red flags and ATTR-CM diagnosis likely reflects increased disease awareness and improved recognition in recent years. The majority of patients in our cohort were diagnosed after 2020 in specialized centers with expertise in cardiac amyloidosis, indicating that once cardiac involvement becomes evident, contemporary diagnostic pathways are now more efficient. Sankey analysis further supports this interpretation, showing that although many patients initially present with noncardiac manifestations, most ultimately reach diagnosis through cardiology referral. This progressive shift in referral pathways highlights persistent gaps in early recognition and the importance of multidisciplinary approach in the diagnostic process. In line with recent real-world studies,^23^ our findings suggest that earlier recognition of extracardiac red flags in populations at higher risk of ATTR-CM is an opportunity not to be missed to reduce significantly the diagnostic delay and enable earlier referral to specialized centers.

In our cohort, the median time from red-flag onset to diagnosis emerged as a clinically interpretable metric with the ability to captures the time interval during which disease-related manifestations are present but are unrecognized. Rather than reflecting diagnostic delay alone, this metric may also capture aspects of disease evolution and patterns of clinical recognition. A longer time from red-flag onset to diagnosis (with a threshold of 9 months) was independently associated with a higher risk of all-cause mortality and CV mortality, even after adjustment for age, sex, NAC disease stage, and tafamidis treatment. Although longer intervals may reflect both delayed clinical recognition and underlying disease evolution, this association suggests that prolonged periods of unrecognized disease manifestations may identify patients at higher risk of adverse outcomes rather than an indolent clinical course. These findings were consistent across age groups, indicating that the prognostic value of this metric may extend to older populations, where traditional markers may be less discriminative.^1^^,^^24^ In contrast, neither the timing of the earliest red flag nor the number of red flags at ATTR-CM diagnosis was associated with outcomes. This suggests that the prognostic value of this metric may lie predominantly in the overall temporal profile through which disease manifestations remain unrecognized before diagnosis rather than their cumulative burden.

The characteristics of our cohort reflect a contemporary ATTR-CM population, with a predominance of early stage disease, relatively low diuretic use, and increasing use of disease-modifying treatments. Compared with historical cohorts such as ATTR-ACT^25^ population, patients included in this study appear to be diagnosed earlier, more closely resembling recent trial populations such as HELIOS-B.^26^ In this context, the association between longer prediagnostic intervals and adverse outcomes suggests that, even in the current era of improved awareness and earlier diagnosis, delays in recognizing prediagnostic manifestations remain clinically meaningful.

These findings have important implications for clinical practice. First, they reinforce the need to move beyond a purely “cardiomyopathy model” of diagnosis and promote broader awareness of extracardiac red flags across specialties, including orthopedics and neurology. Second, they suggest that integrating easily accessible metrics of diagnostic delay into clinical assessment, rather than relying solely on evaluation at diagnosis, may improve risk stratification and decision-making. Notably, as the effect of diagnostic delay is expressed on a per-year basis, even modest increases in risk may translate into a substantial cumulative impact over prolonged periods of unrecognized disease. Finally, in an era of effective disease-modifying therapies, reducing the duration of unrecognized disease represents a key opportunity to improve outcomes and may represent a new quality metric to assess the efficiency of screening strategies at the population level. In this context, structured multidisciplinary approaches such as dedicated interspecialty meetings, hospital-based working groups, and the implementation of integrated care pathways may facilitate earlier recognition of red flags and more timely referral to specialized centers.

### Study Limitations

First, its retrospective design relies on the accuracy and completeness of clinical documentation. The timing of red-flag onset was derived from chart review and may have been affected by variability in reporting or by undocumented manifestations, potentially introducing inaccurate estimates of the true interval between early disease features and diagnosis. Second, the median time from red-flag onset to diagnosis assumes that the first documented manifestation approximates the true onset of that feature, which may not be systematically true. Third, the study is intrinsically subject to survival bias as only patients who survived to receive a diagnosis of ATTR-CM were included, whereas individuals with more aggressive disease phenotypes may have died before diagnosis. Although this reflects real-world clinical practice, such a bias would be expected to attenuate rather than exaggerate the observed association between diagnostic delay and mortality. Fourth, because the cohort was drawn from 2 Italian referral centers with expertise in cardiac amyloidosis, the findings may not be fully generalizable to different settings, where recognition of red flags and referral pathways may differ. As both recruiting centers are cardiology-based, a referral bias toward cardiology-driven diagnostic pathways cannot be excluded, potentially over-representing cardiology referral pathways and underestimating alternative referral routes from other specialties. Fifth, the population consisted almost entirely of patients with wild-type ATTR-CM, limiting generalizability to hereditary ATTR-CM. Finally, the sample size may have limited power to detect modest interaction effects or less common diagnostic trajectories. Overall, these findings should be interpreted as hypothesis-generating and warrant validation in prospective studies.

## Conclusions

Extracardiac red flags frequently precede the diagnosis of ATTR-CM by several years, providing a prolonged window of clinically detectable but often under-recognized disease. A longer time from red-flag onset to ATTR-CM diagnosis, an easily accessible metric in clinical practice, is independently associated with both all-cause and CV mortality. Improving awareness of red flags across specialties and streamlining referral pathways may represent a key opportunity to optimize patient outcomes in the contemporary era of available disease-modifying treatments.

Perspectives **COMPETENCY IN MEDICAL KNOWLEDGE:** In ATTR-CM, red-flag manifestations frequently emerge years before diagnosis, with extracardiac features often preceding cardiac involvement. The interval between red-flag onset and diagnosis provides clinically relevant information beyond conventional assessment at diagnosis and is independently associated with mortality. Delayed recognition of early manifestations represents a missed opportunity for timely diagnosis and treatment.

**TRANSLATIONAL OUTLOOK:** Future studies should evaluate whether systematic screening strategies based on extracardiac red flags and integration of temporal metrics into clinical assessment can reduce diagnostic delay and improve outcomes. Prospective validation in broader and nonspecialist populations is warranted to assess the generalizability and clinical utility of this time-based approach.

## Funding support and author disclosures

Dr Capovilla has received fees for educational activities from Bristol Myers Squibb; and speaking honoraria from Pfizer. Dr Iseppi has received fees for educational activities from Bristol Myers Squibb and Alnylam Pharmaceutical; and speaking honoraria from Pfizer. Dr Moretti has received fees for educational activities and speaking honoraria from AstraZeneca, Bristol Myers Squibb, Sanofi, Chiesi, Pfizer, Bayer, and Alnylam Pharmaceutical. Dr Merlo has received fees from Pfizer, Novartis, and Vifor Pharma; and an unrestricted general research grant on amyloidosis by Pfizer, without any control on intellectual contents. Dr Porcari has received fees for educational activities from Alnylam Pharmaceutical; and speaking honoraria from Bayer, Pfizer, and AstraZeneca. Dr Sinagra has received fees from Biotronik, Boston Scientific, AstraZeneca, and Novartis. All outside the submitted work. All other authors have reported that they have no relationships relevant to the contents of this paper to disclose.
