# Supplementary material for: Clinical Impact of Diagnostic Delay by Red Flags Assessment in Transthyretin Amyloid Cardiomyopathy
Source: JACC Adv. 2026 Sep 9;5(10):103200. doi: 10.1016/j.jacadv.2026.103200 (PMC13699702; doi:10.1016/j.jacadv.2026.103200)
Supplement: Supplementary file 1 [file mmc1.docx]

**Supplemental Material**

**Clinical Impact of Diagnostic Delay by Red-Flags Assessment in Transthyretin Amyloid Cardiomyopathy**

**Tables**

**Supplemental Table 1. Baseline characteristics according to tafamidis exposure during follow-up.**

| **Variable** | **Total population**  **(n = 208)** | **No tafamidis**  **(n=97)** | **With tafamidis**  **(n=111)** | **p-value** |
| --- | --- | --- | --- | --- |
|  |  |  |  |  |
| Age at diagnosis, y (median [Q1–Q3]) | 80 [76-83] | 82 [79-85] | 78 [74-80] | **p<0.001** |
| Male Sex, n (%) | 174 (83.7) | 80 (82.5) | 94 (84.7) | 0.71 |
| ATTR type |  |  |  | 0.45 |
| ATTRwt, n (%) | 201 (96.6) | 95 (97.9) | 106 (95.5) |  |
| ATTRv, n (%) | 7 (3.4) | 2 (2.1) | 5 (4.5) |  |
| Ischaemic heart disease, n (%) | 41 (19.7) | 20 (20.6) | 21 (18.9) | 0.73 |
| Systemic hypertension, n (%) | 166 (79.8) | 77 (79.4) | 89 (80.2) | >0.99 |
| Atrial fibrillation at diagnosis, n (%) | 80 (38.5) | 47 (48.5) | 33 (29.7) | **0.006** |
| N° of red flags per patient (median [Q1–Q3]) | 5 [4-6] | 5 [4-6] | 5 [4-6] | 0.82 |
| Median RF time before diagnosis, months (median [Q1–Q3]) | 9 [4-34] | 9 [4-33] | 8 [4-36] | 0.96 |
| NT-proBNP at diagnosis, pg/ml (median [Q1–Q3]) | 1953 [1001-3761] | 2870 [1461-5153] | 1522 [799-2573] | **p<0.001** |
| eGFR, ml/min/m^2^ (median [Q1–Q3]) | 60 [46-75] | 52 [39-61] | 62 [54-78] | **p<0.001** |
| NAC stage |  |  |  | **p<0.001** |
| NAC stage 1 at diagnosis, n (%) | 113 (59.5)* | 34 (41.5)* | 79 (73.1)* |  |
| NAC stage 2 at diagnosis, n (%) | 56 (29.5)* | 31 (37.8)* | 25 (23.2)* |  |
| NAC stage 3 at diagnosis, n (%) | 21 (11.0)* | 17 (20.7)* | 4 (3.7)* |  |
| Echocardiographic parameters at diagnosis |  |  |  |  |
| IVS thickness, mm (median [Q1–Q3]) | 18 [15-20] | 18 [16-20] | 17 [15-19] | 0.081 |
| LVEF, % (median [Q1–Q3]) | 56 [48-63] | 55 [48-65] | 56 [49-61] | 0.57 |
| GLS, –% (median [Q1–Q3]) | –12.9 [–10,–15] | –11.3 [–10,–14] | –13.7 [–10,–15] | 0.25 |
| TAPSE, mm (median [Q1–Q3]) | 19 [15-21] | 18 [14-19] | 20 [15-23] | **0.005** |
| sPAP, mmHg (median [Q1–Q3]) | 39 [32-48] | 43 [36-51] | 38 [30-46] | **0.003** |
| Loop diuretic dose at diagnosis, mg (median [Q1–Q3]) | 25 [0-62.5] | 50 [25-100] | 25 [0-50] | **p<0.001** |
|  |  |  |  |  |

Abbreviations: ATTRwt = wild-type transthyretin amyloid cardiomyopathy; ATTRv = variant transthyretin amyloid cardiomyopathy; eGFR = estimated glomerular filtration rate; NAC = National Amyloidosis Centre; IVS = interventricular septal; LVEF = left ventricular ejection fraction; GLS = global longitudinal strain; TAPSE = Tricuspid Annular Plane Systolic Excursion; sPAP = Systolic Pulmonary Artery Pressure; RF = Red Flags; HF = heart failure; CV = cardiovascular. *Percentages are calculated among patients with available data for NAC staging. Continuous variables are presented as median (Q1–Q3) and were compared using the Mann–Whitney U test. Categorical variables are presented as number (%) and were compared using the chi-squared or Fisher's exact test, as appropriate.

**Figure Legend**

**Supplemental Figure 1.**

*Panel A*: Kaplan–Meier curves for all-cause mortality stratified by median time from red-flag onset to diagnosis (early vs late groups based on quartiles), using treatment-aligned follow-up. Groups were compared using the log-rank test.

*Panel B*: Cumulative incidence curves for cardiovascular mortality stratified by median time from red-flag onset to diagnosis, accounting for competing risks and using treatment-aligned follow-up. Groups were compared using Gray's test.

In this sensitivity analysis, follow-up was aligned according to treatment status, with time zero defined as tafamidis initiation in treated patients and ATTR-CM diagnosis in untreated patients. Results were consistent with the primary analyses.

*Abbreviations: CV = cardiovascular.*

**Figures**

**Supplemental Figure 1**. **Sensitivity analysis of all-cause and cardiovascular mortality according to median time from red-flag onset to diagnosis using treatment-aligned follow-up.**
